# Death and injuries caused by cattle: A forensic overview

**DOI:** 10.1007/s12024-024-00786-8

**Published:** 2024-01-30

**Authors:** Roger W. Byard

**Affiliations:** School of Biomedicine, The University of Adelaide, Frome Rd, Adelaide, South Australia 5000 Australia

**Keywords:** Cattle, Bull, Cow, Fatality, Forensic, Death, Injury, Farming, Bullfighting, Rodeo

## Abstract

Lethal episodes arising from interactions with cattle may be the result of a response of the animals to simple handling and herding, or from reactions to deliberate provocation or goading intended to incite aggressive behavior for public entertainment purposes. Deaths may be considered to be unprovoked and unanticipated, or provoked and predictable. Cattle cause significant numbers of deaths globally and are considered the most dangerous large animal in Britain. Behavior may be unpredictable even in apparently docile domesticated animals, and attacks may be by a single animal or a herd and result in injuries from kicking, head/butting/charging, stomping, goring, and crushing. Craniofacial injuries may involve fractures of the spine or skull with cerebral contusions and lacerations associated with subarachnoid, subdural, and extradural hemorrhages. Chest injuries are also characterized by fractures which may be multiple with flail chest, hemo- and pneumothoraces, and organ disruption. Injuries to the abdomen and perineum include intestinal perforations, splenic rupture, perineal and vaginal tears, urethral lacerations and avulsions, and bladder and rectal perforations. Significant vascular injuries include complete and partial transections and lacerations. Males living in rural areas are most at risk of a fatal encounter.

## Introduction

There are a number of ways that death can be caused by animals that include blunt and sharp force injuries most often arising from crushing, stomping, goring, or biting [1]. Other deaths may result from envenomation or poisoning [2]. While mechanisms and circumstances of death have been studied for a range of animals, this has not occurred in a medicolegal context for cattle and so the following review was undertaken as there are some quite unique aspects of the handling of cattle that differ from other domestic species.

## Materials and methods

A literature search was undertaken for the terms cattle, cow, bull, forensic, injury, and death on the PubMed and Google Scholar databases. In addition, these terms were searched for on Google to capture media reports of such events. The search was not aimed at documenting all such cases, merely to determine the range of activities that may lead to serious injuries or death related to the handling of, or association with, cattle.

## Discussion

### Overview

Deaths involving cattle are slightly unusual when compared to fatalities related to other species, as lethal events may occur not only from handling and herding these animals but also from deliberate provocation or goading to initiate aggressive behavior, as in bullfights and rodeos. Thus, deaths may be unprovoked and unanticipated (e.g., during farm work), or provoked and somewhat predictable (e.g., during a bullfight).

### Farming

Cattle are responsible for a significant number of deaths in agricultural areas globally. This is due to several factors related to the sheer number that are raised for meat and milk and also to their size which may make handling difficult. They may also be unpredictable in their behavior and can move quickly [3, 4]. The force from kicks, stomping, or head butting [4, 5], similar to that from horses, donkeys, and camels [6–8], can cause devastating lethal blunt force head, chest, and abdominal injuries. Although kicking is more common, head butt/charging and trampling are associated with more severe injuries [9]. Crushing in a confined space may also happen when cattle are being herded into stalls or onto trucks for transport. As with water buffalo, goring from horns may occur resulting in deep vascular, soft tissue, and organ injuries [10]. While the majority of these deaths are uncomplicated, on occasion, lethal trauma from animal attacks has initially been confused with homicide [11, 12].

The majority of the 1610 animal-related fatalities in the United States occurring between 2008 and 2015 were due to “other mammals” which consisted predominantly of horses and cattle [13]. It has been estimated that approximately 20–22 deaths occur each year in the United States from cattle, only 10 of which involved bulls. In five cases, multiple cows were involved in the attack, usually when walkers, joggers, or cyclists had entered pastures. The presence of a dog may provoke an attack [14]. In the United Kingdom, 74 people were killed by cattle (70% due either to bulls or newly calved cows). This has led cattle to be declared the most dangerous large animal in Britain [15, 16].

Farm, transport, and abattoir workers are most at risk from injuries and death caused by domestic cattle [17]. In Victoria, Australia, for example, cattle handling is one of the top three causes of deaths on farms [3] with farming being the sixth most dangerous occupation in the United States [18] and the most dangerous occupation in Ireland [19]. If hours of exposure are taken into account, the risk of a bull-related death is higher than that associated with the use of a tractor [20].

### Types of injuries

Typical chest injuries include multiple rib fractures with flail chests, hemo- and pneumothoraces, and organ damage. Abdominal injuries often involve goring and include intestinal perforations and liver and splenic lacerations [21]. Perineal injuries may also occur [22, 23] ranging from simple vulval abrasions and hematomas to complicated perineal and vaginal tears, urethral lacerations and avulsions, and bladder and rectal perforations [24]. Craniofacial injuries can arise from trampling or kicking [25] and include spinal or skull fractures, subdural, subarachnoid, extradural hemorrhages, and cerebral contusions and lacerations. Vascular injuries usually occur in the perineum or legs and range from complete to partial vessel transection or laceration. Intimal tears may be associated with subsequent thrombus formation [26]. Those most at risk of such injuries include males and the elderly living in rural areas [27]. Underlying comorbidities and treatments such as anticoagulation may exacerbate the effects of these injuries in older individuals [5].

A study from Poland showed that 98 of 1872 animal-related injuries were due to bulls, with 92 (94%) of the cases occurring in rural areas [28]. Rarely, however, goring injuries involving children have been reported [29]. Activities associated with deaths include working in an enclosed space (33%), herding or moving (24%), loading (14%), or feeding cattle (14%) [15]. In addition to these potential dangers, cattle mustering, particularly on large properties such as those found in outback Australia, may be associated with accidents involving vehicles such as quad bikes which may be unstable in irregular terrain [30].

Other transport-related injuries and deaths may occur with cattle which may involve single vehicle/motor cycle crashes or larger scale incidents where trains have been derailed. This has occurred in a number of countries when trains have collided with cattle on the track. In these cases, it is usually the cattle who suffer injury and death [31–33]. In a highly unusual case in India, a man who was standing near a rail track was killed when a cow that had been hit by a train was launched into the air and impacted him [34].

### Bullfighting (La Corrida)

One of the more unusual aspects of fatalities associated with cattle relates to recreational activities where bulls are encouraged and expected to act aggressively. This may take the form of bullfighting, running with bulls, or riding bulls in rodeos. The divergence of opinion on bullfighting is well captured by quotes from two great American writers: while Steinbeck stated that “I think bullfights are for men who aren’t very brave and wish they were,” this contrasted (somewhat predictably) with Hemingway who considered that “Bullfighting is the only art in which the artist is in danger of death and in which the degree of brilliance in the performance is left to the fighter’s honor.” It is estimated that approximately 1600 bullfighting events occur each year in Spain with different formats including variable ages of the bulls, and fighting on foot or horseback [35].

**Fig. 1:**
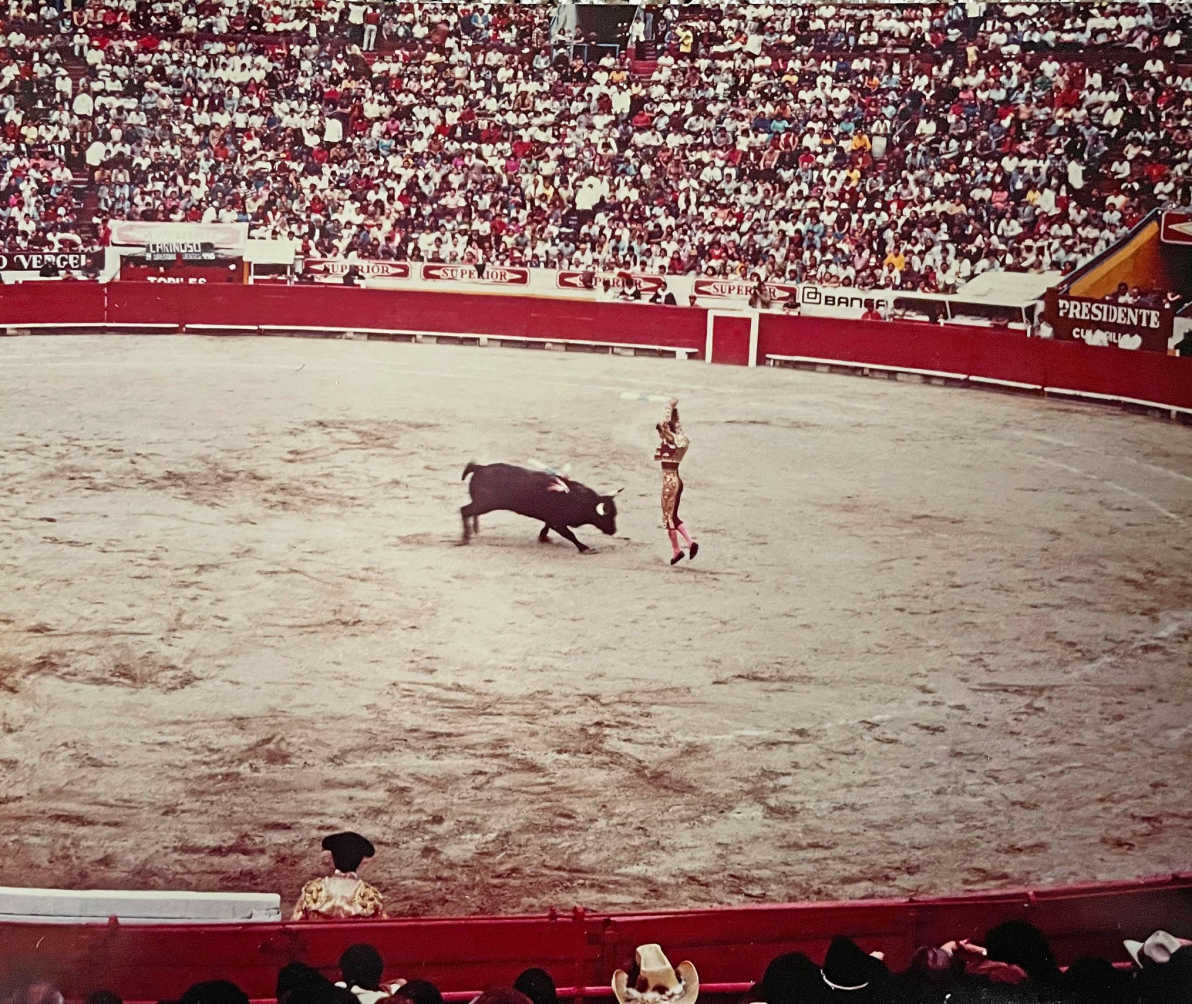
A *banderillero* stabbing a bull in the neck with pointed darts during a bullfight at the Plaza de Toros México in Mexico City

Bulls used for fighting, *toro brava*, are generally the largest and most aggressive and weigh in between 500 and 730 kg (1100–1600 lbs). They undergo minimal handling and are essentially raised as “a wild animal on special farms” [36]. The fight is staged with *picadors* initially spearing the bull’s neck muscles so that its forward movement and tossing muscles are weakened, therefore preventing it from raising its head. The *banderillas* then add to the injury by stabbing with short barbed spears [36, 37] (Fig. 1). Approximately 180,000 bulls are killed in bullfights each year globally [38]. The activity is not, however, without risk of injury or death for the matador [39], with a study that examined cases from Spain, Portugal, and southern France showing a mean accident rate of 9.13% and a mortality rate of 0.48%.

The most common injury involves damage to blood vessels in the thigh and groin from goring, with prognostic factors for a lethal outcome in the ring being vascular injuries and goring in the back [35]. Deep perineal injuries may also be associated with serious vascular damage [37]. Other injuries may be caused by impact from charging, falling, or stomping. If a bullfighter is facing the bull, injuries are most likely in the upper internal thigh and lower abdominal quadrants. This contrasts with situations where a bullfighter might be attempting to run from a bull where injuries occur more often in the buttocks, posterior thighs, and perineum [39]. If horn penetration is deep enough, a bull can then lift a bullfighter into the air by extending the neck. Rotational head movements may then cause extensive destruction of deep tissues with marked bacterial contamination [39]. Angular acceleration following being lifted and thrown also increases the risk of blunt cranio-cervical trauma on impact with the ground [36].

### Running of the bulls

Bulls also feature prominently in certain festivals, particularly in Spain. The most well-known fiesta involving bulls is probably the Running of the Bulls in Pamplona in July, made famous by Hemingway’s book *The Sun Also Rises*. While hundreds of mainly minor injuries are dealt with each year, 16 people have died since 1910. Deaths are mostly caused by goring, trampling, or blunt trauma from impact with horns [40]. A case of crush asphyxia beneath a pile of fallen runners has been reported [41]. More deaths have, however, occurred in other festivals, with greater than 30 fatalities claimed to have occurred in Valencia’s bull-running events since 2015 [42].

An analysis of injuries caused by bull horns at fiestas that were treated at a single center in Spain over a 41-year period involved a total of 296 patients. The mean age was 33.4 years (range 17–91 years) with 288 males (97.3%). The mortality was 5.1% with risk factors being arterial injuries, abdominal injuries, and an age over 65 years [43].

### Rodeos

Another situation where bulls are goaded into performing for crowds involves rodeos. Rodeos have been described as one of the most dangerous sports in the world with data showing that approximately 20 of every 100,000 rodeo contestants suffered severe injury compared to the rate among football players of less than one in every 100,000. Stomping on the chest or back has been associated with the worst outcomes. Bull riding had the greatest injury frequency with 16 deaths on the bull riding circuit occurring between 1989 and 2009 [44–46]. Deaths have occurred in riders who have fallen while bull riding and been kicked by the animal’s back legs, despite the wearing of protective head and chest equipment [47]. Fatalities at rodeos have involved children [48] and have also occurred in participating animals including bulls, steers, cows, and calves [49].

## Conclusion

The size and unpredictable nature of cattle make them potentially dangerous to work with. Lethal injuries may result from both blunt and sharp force trauma and may occur unexpectedly in farming, or during activities such as bullfighting and rodeos where the animals are provoked to behave aggressively. Very characteristic injuries from trampling, crushing, and goring may all be found during the autopsy evaluation of such cases.

## Key points

1. Deaths caused by cattle may result from simple handling and herding, or from deliberate provocation or goading.
2. Injuries result from kicking, head/butting/charging, stomping, goring, and crushing.
3. Blunt and sharp force trauma may injure the head, chest, abdomen, and limbs.
4. Males living in rural areas involved in farming activities are most at risk of a fatal encounter.

## References

[1] Bury D, Langlois N, Byard RW. Animal-related fatalities—Part I: Characteristic autopsy findings and variable causes of death associated with blunt and sharp trauma. J Forensic Sci. 2012;57:370–4. 21981339 10.1111/j.1556-4029.2011.01921.x

[2] Bury D, Langlois N, Byard RW. Animal-related fatalities—Part II: Characteristic autopsy findings and variable causes of death associated with envenomation, poisoning, anaphylaxis, asphyxiation, and sepsis. J Forensic Sci. 2012;57:375–80. 21981407 10.1111/j.1556-4029.2011.01932.x

[3] WorkSafe Victoriaaz. Worker fatally injured in cattle yard incident. 2023. https://aus01.safelinks.protection.outlook.com/?url=https%3A%2F%2Fwww.worksafe.vic.gov.au%2Fsafety-alerts%2Fworker-fatally-injured-cattle-yard-incident&data=05%7C01%7C. Accessed 10 Dec 2023.

[4] Grealish S. Terrifying moment farmer narrowly escapes being trampled by a rampaging cow as it headbutts her in horrifying clip. The Sun. 2023. https://www.thesun.co.uk/news/22062194/terrifying-moment-farmer-attacked-cow/. Accessed 10 Dec 2023.

[5] Yamamoto Y, Aoki Y. Domestic cow-related severe facial trauma in an older farmer undergoing anticoagulation treatment. Cureus. 2022;14:e29818. 36337827 10.7759/cureus.29818 PMC9626379

[6] Byard RW. Lethal recreational activities involving horses – A forensic study. Leg Med. 2020;46:101728. 10.1016/j.legalmed.2020.101728 32531668

[7] Fogel L, Varga G, Hubay M, Felszeghy E, Varga P, Byard RW. Autopsy features of fatal donkey attack. Am J Forensic Med Pathol. 2018;39:354–6. 29727315 10.1097/PAF.0000000000000406

[8] Gilbert JD, Byard RW. Camel-related deaths – A forensic overview. Am J Forensic Med Pathol. 2021;42:46–50. 32925211 10.1097/PAF.0000000000000606

[9] Murphy CG, Cow-related trauma,. A 10 year review of injuries admitted to a single institution. Injury. 2010;41:548–50. 19729160 10.1016/j.injury.2009.08.006

[10] Byard RW. Causes and mechanisms of death in fatal water buffalo attacks. J Forensic Sci. 2017;62:934–6. 28066906 10.1111/1556-4029.13358

[11] Gudmannsson P, Berge J, Druid H, Ericsson G, Eriksson A. A unique fatal moose attack mimicking homicide. J Forensic Sci. 2018;63:622–5. 28631272 10.1111/1556-4029.13579

[12] Murray LA, Sivaloganathan S. Rambutt - The killer sheep. Med Sci Law. 1987;27:95–7. 3586946 10.1177/002580248702700205

[13] Forrester JA, Weiser TG, Forrester JD. An update on fatalities due to venomous and nonvenomous animals in the United States (2008–2015). Wild Environmental Med. 2018;29:36–44. 10.1016/j.wem.2017.10.004 29373216

[14] Rhind JH, Quinn D, Cosbey L, Mobley D, Britton I, Lim JJ. Cattle-related trauma: A 5-year retrospective review in a adult major trauma center. Emerg Trauma Shock. 2021;14:86–91. 10.4103/JETS.JETS_92_20 PMC8312910 34321806

[15] Van Niekerk T. How many people are killed by cows each year. World Animal Foundation. 2023. https://worldanimalfoundation.org/advocate/how-many-people-killed-by-cows/. Accessed 10 Dec 2023.

[16] Pemberton A, Peacock A. Man killed and wife left paralysed after being trampled by a herd of cows as they walked dogs. Mirror. https://www.mirror.co.uk/news/uk-news/man-killed-wife-left-paralysed-28967626. Accessed 10 Dec 2023.

[17] Byard RW. Farming deaths – An ongoing problem. Forensic Sci Med Pathol. 2017;13:1–3. 28091983 10.1007/s12024-017-9839-8

[18] Proctor R, Leonard M, Lawson C, Linh H, Quinn M, Burns B Jr. Impact of injuries on hospital resource utilization among trauma patients admitted due to accidents caused by farm animals. Cureus. 2020;12: e8270. 32596086 10.7759/cureus.8270 PMC7314374

[19] Sheehan M, Deasy C. A descriptive study of the burden of animal-related trauma at Cork University Hospital. Ir Med J. 2018;111:673. 29869854

[20] Sheldon KJ, Deboy G, Field WE, Albright JL. Bull-related incidents: Their prevalence and nature J Agromedicine. 2009;14:357–69. 19657885 10.1080/10599240903042024

[21] Dogan KH, Demirci S, Erkol Z, Sunam GS, Kucukkartallar T. Injuries and deaths occurring as a result of bull attacks. J Agromedicine. 2008;13:191–6. 19064423 10.1080/10599240802405975

[22] Okur MI, Yildirim AM, Köse R. Severe haematoma of the vulva and defloration caused by goring. Eur J Obstet Gynecol Reprod Biol. 2005;119:250–2. 15808390 10.1016/j.ejogrb.2004.02.047

[23] Maity S, Puranik A, Baskaran S, et al. Patterns and management of unprovoked bull attack injuries: A retrospective case series. Cureus. 2022;14: e33075. 36721567 10.7759/cureus.33075 PMC9883671

[24] Priyadarshi V, Gupta D, Pal DK. Lower genitourinary tract trauma caused by cow horn injury. J Obstet Gynaecol India. 2016;66(Suppl 1):578–82. 27651664 10.1007/s13224-015-0748-z PMC5016394

[25] Zhang QB, Zhang B, Zhang ZQ, et al. The epidemiology of cranio-facial injuries caused by animals in southern-central China. J Craniomaxillofac Surg. 2012;40:506–9. 21925891 10.1016/j.jcms.2011.08.012

[26] Bhatt S, Vaidya S, Karmacharya RM, Tamang A, Manandhar A, Neupane M. Circumferential intimal tear with thrombosis of right superficial femoral artery due to penetrating injury by bull horn: A case report. Ann Med Surg (Lond). 2022;74:103228. 35127064 10.1016/j.amsu.2021.103228 PMC8792411

[27] Shriyan SV, Mani UA, Bhot FB, Sada EC, Ursekar R, Adake D. Animal injuries; A case series of bull induced injuries in India. Adv J Emerg Med. 2019;4:e5. 31938774 10.22114/ajem.v0i0.244 PMC6955030

[28] Nogalski A, Jankiewicz L, Cwik G, Karski J, Matuszewski L. Animal related injuries related at the Department of Trauma and Emergency Medicine, Medical University of Lublin. Ann Agric Environ Med. 2007;4:57–61. 17655178

[29] Casaní-Martinez C, Morales-Suárez-Varela M,. Bull horn lesions in childhood (Letter). Pediatrics. 2000;105:685–6. 10733400 10.1542/peds.105.3.685

[30] Radford T. PS News. WorkSafe investigating fatal cattle mustering incident north of Perth. 2023. https://aus01.safelinks.protection.outlook.com/?url=https%3A%2F%2Fpsnews.com.au%2Fworksafe-investigating-fatal-cattle-mustering-incident-north-of-perth%2F120095%2F&data=05%7C01%7C. Accessed 10 Dec 2023.

[31] Rawat M. Train derails in Kerala after hitting cattle on track. Times Now https://www.timesnownews.com/india/nilambur-road-shoranur-express-special-train-derails-in-kerala-after-hitting-cattle-on-track-article-105240593#:~:text=New%20Delhi%3A%20In%20Kerala%2C%20train,or%20injuries%20have%20been%20reported. Accessed 10 Dec 2023.

[32] Bullen J. Train derailed after 70mph smash into cattle – Despite warning of cattle on the line. The Standard 2016 https://www.standard.co.uk/news/transport/train-derailed-after-70mph-smash-into-cattle-despite-warning-of-cows-on-the-line-a3219361.html#:~:text=A%20train%20derailed%20after%20it,Ashford%20at%20around%209.40pm. Accessed 10 Dec 2023.

[33] NZ Herald. Train derails after hitting cattle. https://www.nzherald.co.nz/nz/train-derails-after-hitting-cattle/XDBRFMRAZMIWMS33UDLURX7LNM/. Accessed 10 Dec 2023.

[34] Hooper S. Man killed by a flying cow that was hit by a train and flung 100ft in freak accident while urinating on tracks 2023 https://www.thesun.co.uk/news/22123893/flying-cow-death-accident-india-train-freak/. Accessed 10 Dec 2023.

[35] Reguera-Teba A, Martínez-Casas I, Torné-Poyatos P, Hernández-Cortés P. Eight-year analysis of bullfighting injuries in Spain. Portugal and southern France Sci Rep. 2021;11:16006. 10.1038/s41598-021-94524-7 PMC8346594 34362939

[36] Spiotta AM, Matoses SM. Neurosurgical considerations after bull goring during festivities in Spain and Latin America. Neurosurgery. 2011;69:455–61. 21792140 10.1227/NEU.0b013e3182191fb1

[37] Rudloff U, Gonzalez V, Fernandez E, Holguin E, Rubio G, Lomelin J, et al. Chirurgica Taurine: A 10-year experience of bullfight injuries. J Trauma. 2006;61:970–4. 17033570 10.1097/01.ta.0000196871.19566.92

[38] Humane Society International/Global. Bullfighting: A long, cruel death. https://www.hsi.org/news-resources/bullfighting-long-cruel-death/. Accessed 10 Dec 2023.

[39] García-Marín A, Turégano-Fuentes F, Sánchez-Arteaga A, Franco-Herrera R, Simón-Adiego C, Sanz-Sánchez M. Bullhorn and bullfighting injuries. Eur J Trauma Emerg Surg. 2014;40:687–91. 26814783 10.1007/s00068-014-0381-z

[40] BBC. Bull gores man to death in Spain. 2009. http://www.bbc.co.uk.

[41] Running of the bulls deaths. https://www.runningofthebulls.com/history-of-the-bulls/running-of-the-bulls-deaths/. Accessed 10 Dec 2023.

[42] CBS News. With 10 people killed this summer alone, could Spain say adios to bull-running festivals? 2022. https://www.cbsnews.com/news/spain-bull-running-festivals-10-deaths-fuel-debate/. Accessed 10 Dec 2023.

[43] San Norberto EM, Martín-del Olmo JC, Diago MV, Taylor JH, Vaquero C. Bull horn vascular injuries in popular celebrations: A 40-year retrospective analysis. J Vasc Surg. 2022;75:2030–6. 35063613 10.1016/j.jvs.2022.01.009

[44] Butterwick DJ, Hagel BH, Meeuwisse WH. Epidemiologic analysis of injury in five years of Canadian professional rodeo. Am J Sports Med. 2002;30:193–8. 11912087 10.1177/03635465020300020801

[45] Butterwick DJ, Lafave M, Lau B, Freeman T. Rodeo catastrophic injuries and registry: Initial retrospective and prospective report. Clin J Sport Med. 2011;21:243–8. 21430525 10.1097/JSM.0b013e318218acdd

[46] Graveland. B. Rodeo one of the most dangerous sports in the world, study finds. https://www.theglobeandmail.com/news/national/rodeo-one-of-the-most-dangerous-sports-in-the-world-study-finds/article586921/. Accessed 10 Dec 2023.

[47] Dennis J. Rodeo rider dies after being kicked by bull in Warwick, in Queensland’s Southern Downs. 2023. https://www.abc.net.au/news/2023-01-01/warwick-rodeo-death-queensland-bullrider/101820032. Accessed 10 Dec 2023.

[48] Martinez G. 14-year-old bull rider dies in North Carolina rodeo accident. 2023. https://www.cbsnews.com/news/denim-bradshaw-dies-age-14-bull-rider-north-carolina-rodeo/. Accessed 10 Dec 2023.

[49] Rust S. California rodeo animals face violent and deadly casualties: Broken backs, legs and skulls. Los Angeles Times 2022 https://www.latimes.com/california/story/2022-12-07/rodeo-casualties-for-animals-that-are-ridden-and-roped-broken-backs-legs-and-skulls. Accessed 10 Dec 2023.

